# Colchicine reduces inflammatory cytokines and improves symptoms in HFpEF: an observational pilot study

**DOI:** 10.3389/fmed.2025.1702293

**Published:** 2026-01-16

**Authors:** Meijing Shi, Xinyue Zhang, Lizhuo Li, Yu Wang, Qingzhen Zhao, Yuzhi Zhen, Yaomeng Huang, Chao Liu

**Affiliations:** 1 School of Graduate, Hebei Medical University, Shijiazhuang, China; 2 Department of Cardiology, The First Affiliated Hospital of Hebei Medical University, Shijiazhuang, China; 3 Laboratory Center, The First Affiliated Hospital of Hebei Medical University, Shijiazhuang, China

**Keywords:** colchicine, heart failure with preserved ejection fraction, inflammatory cytokines, observational pilot study, symptoms

## Abstract

**Objective:**

The objective of this study was to investigate whether colchicine could safely and effectively reduce inflammatory marker levels and improve the prognosis in patients with heart failure with preserved ejection fraction (HFpEF).

**Methods:**

This study enrolled patients diagnosed with HFpEF. Venous blood samples were collected to assess the levels of inflammatory markers such as IL-6, IL-8, and TNF-α. If these markers were elevated, patients were treated with colchicine 0.5 mg once daily. Inflammatory markers were reassessed after 2 weeks of treatment in the outpatient department. The primary endpoint was the change in inflammatory factor levels before and after colchicine treatment. Secondary outcomes included assessments of anxiety (HAMA), depression (HAMD), and quality of life (KCCQ) after 1 month of colchicine treatment. The clinical trial was registered at Chinese Clinical Trail Registry (ChiCTR2500103522).

**Results:**

A total of 126 patients were included. The serum inflammatory markers most notably elevated in the HFpEF cohort were IL-8 and IL-6, with IL-8 showing the most significant increase. After 2 weeks of colchicine treatment, serum levels of inflammatory markers decreased significantly. IL-8 levels decreased by −28.65 (95% CI: −54.20 to −11.47, *p* = 0.0010), a 65.5% reduction (95% CI: −82.0% to −45.0%). IL-6 levels decreased by −1.925 (95% CI: −4.510 to −0.29, *p* = 0.0028), reflecting a 30.5% reduction (95% CI: −54.0% to −5.0%). After 1 month of colchicine treatment, patients’ overall quality of life improved significantly. Anxiety (HAMA) decreased by −1 (95% CI: −1.0 to −1.0, *p* < 0.0001), depression (HAMD) decreased by −1 (95% CI: −1.0 to 0.0, *p* < 0.0001), and quality of life (KCCQ) increased by 10 points (95% CI: 9.0 to 12.0, p < 0.0001).

**Conclusion:**

This study is the first to explore colchicine as an anti-inflammatory treatment for HFpEF. Our results indicate that colchicine can safely and effectively reduce inflammatory markers such as IL-8 and IL-6. After 1 month of treatment, significant improvements were observed in anxiety, depression, and quality of life. These findings support colchicine’s potential as a therapeutic option for managing inflammation and improving outcomes in patients with HFpEF.

## 1 Introduction

Heart failure (HF) is a multifactorial clinical syndrome resulting from structural or functional impairments of the heart, and it represents the final stage of various cardiac diseases (1). Among the different types of heart failure, heart failure with preserved ejection fraction (HFpEF) accounts for at least 50% of all cases, and its prevalence is steadily increasing, making it the most common form of heart failure (2, 3). Emerging evidence indicates that multiple inflammatory mediators, including IL-1β, IL-6, TNF-α, and others, along with their associated signaling pathways, play pivotal roles in myocardial remodeling, endothelial dysfunction, and diastolic impairment in HFpEF, with elevated circulating levels correlating closely with disease severity, clinical manifestations, and adverse prognosis (4–8).

Colchicine is a well-established anti-inflammatory agent whose primary mechanisms of action include disruption of microtubule function, inhibition of selectin expression, reduction of neutrophil–platelet interactions, and suppression of NLRP3 inflammasome activation, thereby downregulating the release of inflammatory cytokines such as IL-1β and IL-6 (9, 10). Clinically, colchicine has established applications in gout, familial Mediterranean fever, and other inflammatory cardiovascular conditions. In addition, low-dose colchicine is commonly used for the prevention of recurrent pericarditis and as secondary prevention in patients with coronary artery disease, demonstrating favorable safety and efficacy in large clinical trials. Common adverse effects include mild gastrointestinal symptoms and liver function abnormalities, with rare occurrences of myelosuppression; caution is advised when used at high doses or in patients with impaired renal function (11).

Given the pivotal role of inflammation in the pathogenesis of HFpEF and the limited evidence regarding the anti-inflammatory potential of colchicine in this population, this study proposes the novel use of colchicine as an anti-inflammatory therapy for HFpEF. We comprehensively evaluated multiple cytokines related to inflammatory pathways to explore whether colchicine can safely and effectively reduce systemic inflammation and improve short-term clinical outcomes in these patients.

## 2 Methods

### 2.1 Trial design

This was a single-center, single-arm, prospective interventional study conducted at the First Affiliated Hospital of Hebei Medical University. A total of 126 patients with HFpEF were enrolled from June to August 2025. The study included measurements of inflammatory markers in the venous blood of patients, and if elevated levels of these markers were detected, patients received colchicine 0.5 mg daily. Follow-up assessments were performed accordingly. The study protocol and data analysis were approved by the Ethics Committee of the First Affiliated Hospital of Hebei Medical University in accordance with the Declaration of Helsinki (Approval number: 2025-072-01). Written informed consent was obtained from all participants prior to enrollment. The clinical trial was registered at Chinese Clinical Trail Registry (ChiCTR2500103522).

### 2.2 Trial population

Inclusion criteria were as follows:

- Diagnosed with HFpEF: The diagnosis was based on hemodynamic measurements obtained through left heart catheterization, which is considered the gold standard for HFpEF diagnosis (12). Resting left ventricular end-diastolic pressure (LVEDP) > 15 mmHg, or clinical diagnosis was further supported by a straight leg raising test, with a LVEDP ≥19 mmHg when LVEDP was measured to be >10 mmHg but ≤15 mmHg at rest.
- Elevated inflammatory markers: Patients with one or more inflammatory markers above the normal range were included.

Exclusion criteria included:

- Echocardiographic evidence of an ejection fraction <50%.
- Patients under 18 years of age.
- Evidence of myocardial ischemia on coronary angiography that required interventional therapy.
- Congenital heart disease, restrictive cardiomyopathy, pulmonary arterial hypertension.
- Severe hepatic or renal dysfunction, advanced malignancy, autoimmune disease, multi-organ failure, or any other condition that might interfere with the study outcomes.

### 2.3 Trial procedures

Eligible participants were administered colchicine at a dose of 0.5 mg once daily. Inflammatory marker levels were reassessed after 2 weeks of treatment at the outpatient clinic. Additionally, quality of life assessments were performed before and after 1 month of treatment.

### 2.4 Cytokine measurements

Venous blood samples were collected at baseline and after 2 weeks of treatment, with cytokine levels measured on the same day. All assays were performed using cytokine detection kits based on magnetic particle luminescence (Su, 20,220,256) according to the manufacturer’s instructions. Each sample was measured in duplicate, and the mean value was used for analysis. Standard curves were generated for each assay, and intra- and inter-assay coefficients of variation were strictly maintained below 10%. Negative and positive controls were included to ensure the accuracy and reproducibility of the measurements.

### 2.5 Trial endpoints

The primary efficacy endpoint was the change in inflammatory factor concentrations from baseline to 2 weeks. Exploratory endpoints included the assessment of patient symptoms using the Hamilton Anxiety Scale (HAMA), the Hamilton Depression Scale (HAMD), and the Kansas City Cardiomyopathy Questionnaire (KCCQ). Safety endpoints of particular interest included the evaluation of gastrointestinal reactions, liver function, and renal function.

### 2.6 Statistical analysis

Statistical analyses were performed using SPSS 26.0 software. Quantitative data that were normally distributed were expressed as mean ± standard deviation (SD), while non-normal data were presented as median (first, third quartile). Paired t-tests were used for comparisons of data between baseline and follow-up. Statistical significance was set at *p* < 0.05.

## 3 Results

### 3.1 Baseline characteristics

**Figure 1:**
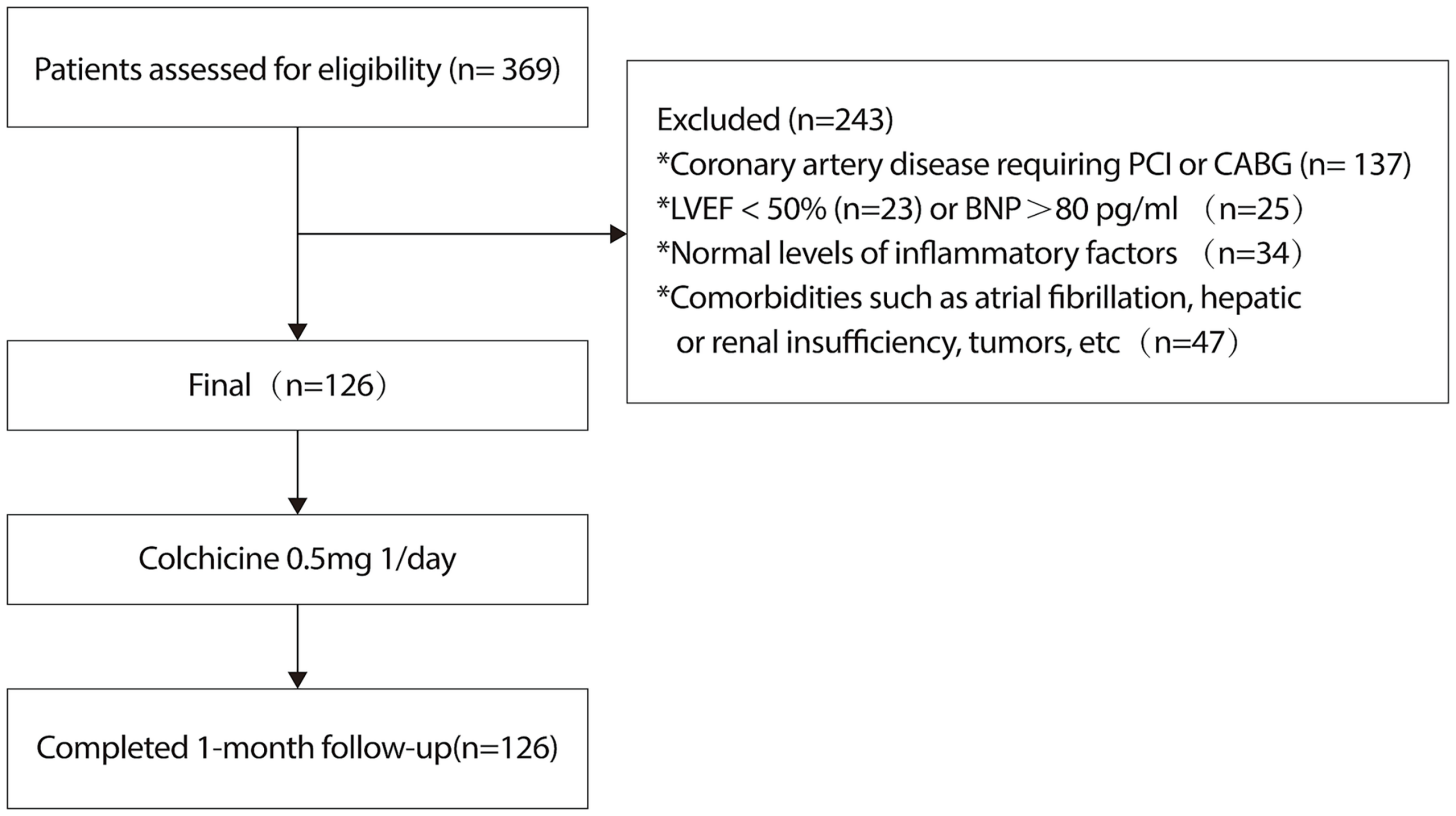
The flowchart shows the enrolment pathway in this study.

During the study period, 365 subjects underwent left heart catheterization and inflammatory marker assessment at the First Hospital of Hebei Medical University, of whom 126 met the inclusion criteria (Figure 1). The baseline clinical characteristics of the study population are summarized in Table 1.

**Table 1:** Baseline characteristics of the patients.

Characteristics	Overall
Age, mean (SD), y	60.45 (±10.20)
Male, No. (%)	65 (60%)
BMI, mean (SD), (Kg/m^2^)	25.59 (±3.39)
SBP (mmHg)	128 (122, 144)
DBP (mmHg)	79.5 (72.25, 88.75)
Hypertension, No. (%)	68 (63%)
Diabetes, No. (%)	21 (19%)
Dyslipidemia, No. (%)	56 (52%)
Atrial fibrillation, No. (%)	10 (9%)
Other types of arrhythmia, No. (%)	23 (21%)
Treatment	
Diuretic agent, No. (%)	29 (27%)
MRA, No. (%)	33 (31%)
ACEI/ARB, No. (%)	4 (4%)
Beta-blocker, No. (%)	66 (61%)
CCB, No. (%)	25 (23%)
ARNI, No. (%)	38 (35%)

BMI, Body Mass Index; SBP, Systolic Blood Pressure; DBP, Diastolic Blood Pressure; MRA, Mineralocorticoid Receptor Antagonists; ACEI, Angiotensin-Converting Enzyme Inhibitors; ARB, Angiotensin II Receptor Blockers; CCB, Calcium Channel Blockers; ARNI, Angiotensin Receptor-Neprilysin Inhibit.

**Figure 2:**
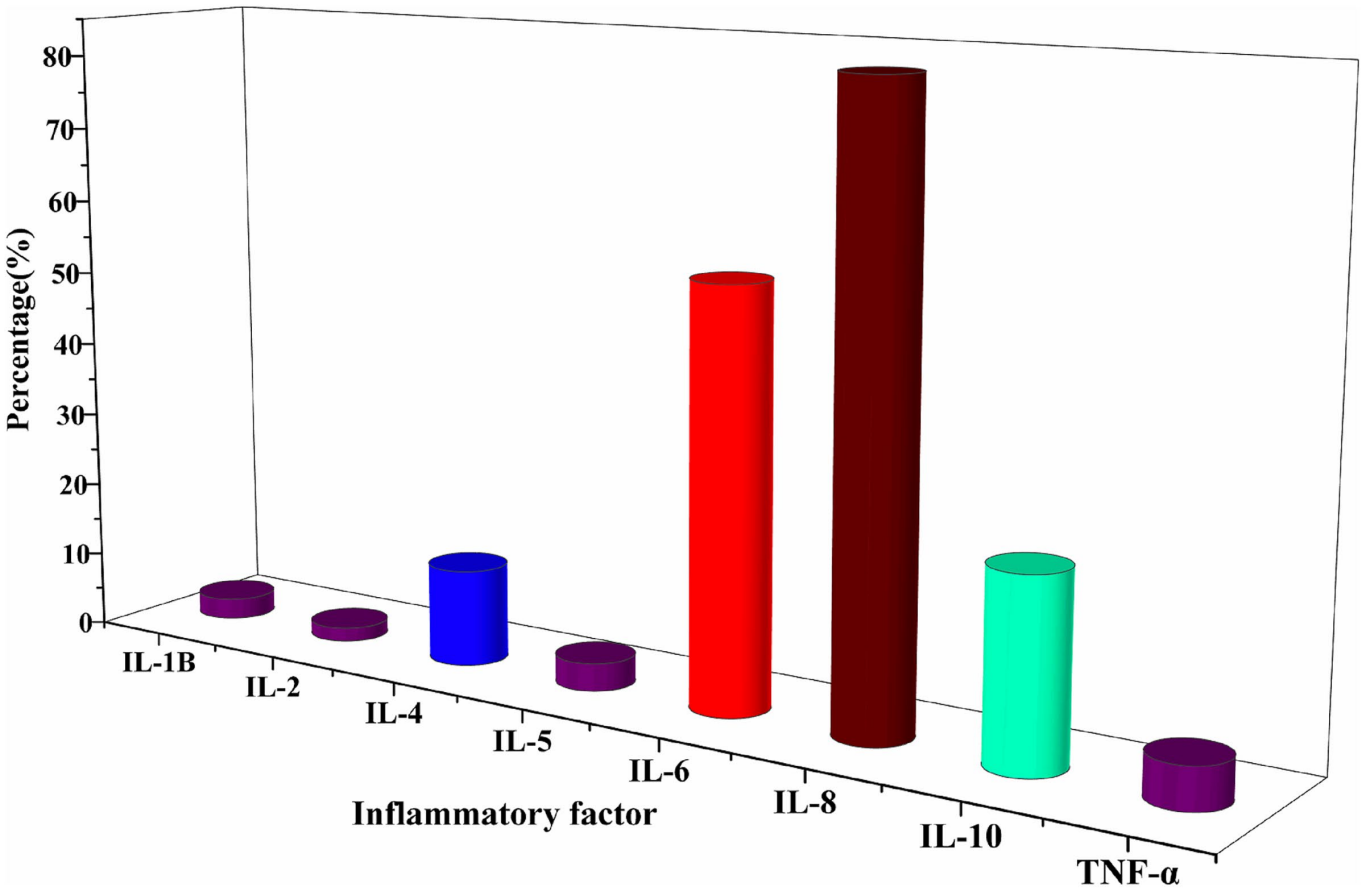
Characterised by elevated levels of inflammatory factors at baseline. The primary inflammatory markers assessed were IL-8 and IL-6.

Among the enrolled HFpEF patients, IL-8 and IL-6 were the primary inflammatory markers analyzed. Both cytokines showed elevated levels in a majority of patients, with IL-8 demonstrating the most pronounced increase (Figure 2; Supplementary Table 1).

### 3.2 Primary efficacy outcome

**Figure 3:**
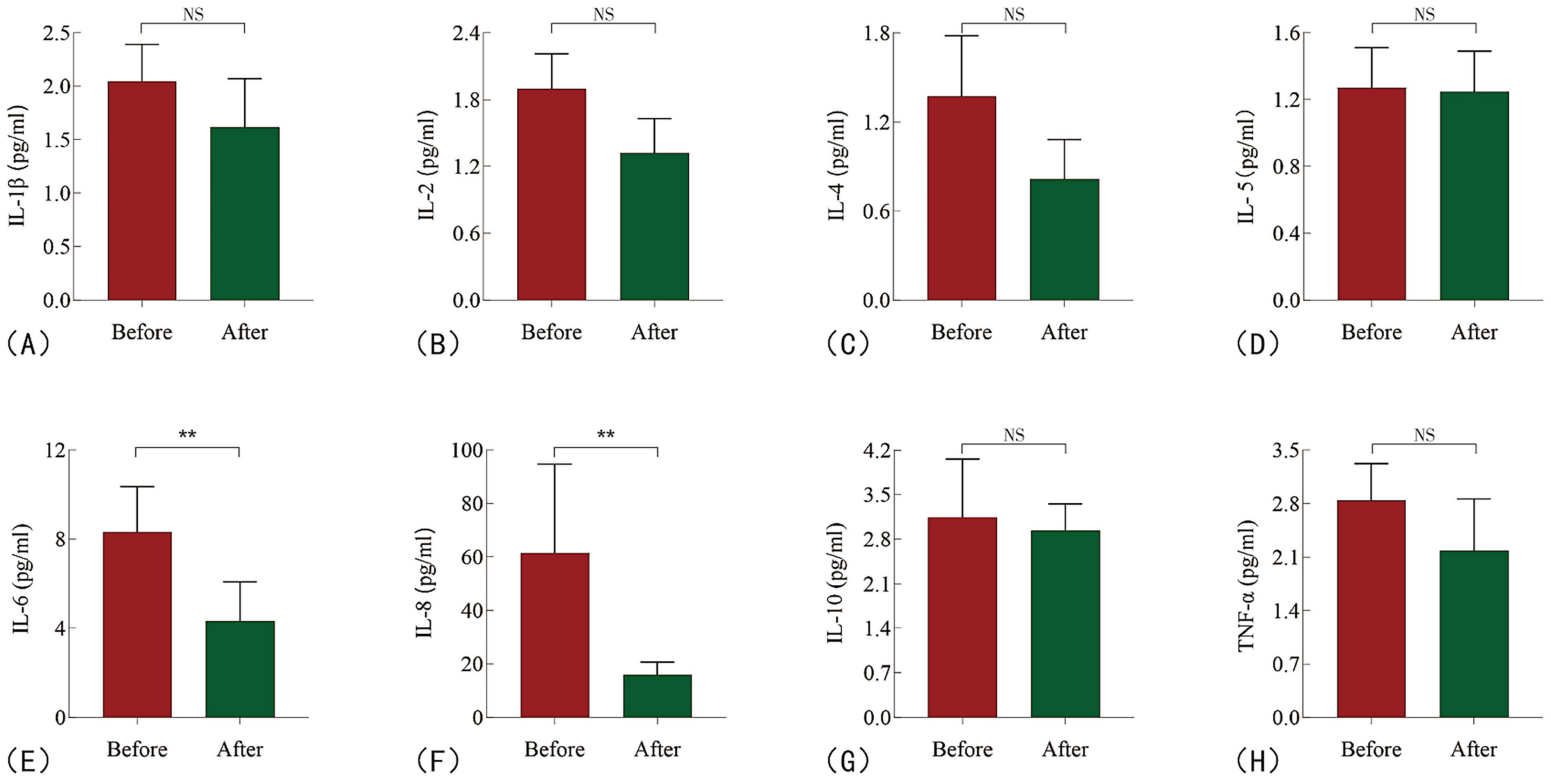
Changes in inflammatory cytokine levels in HFpEF patients before and after treatment. **(A)** IL-1β; **(B)** IL-2; **(C)** IL-4; **(D)** IL-5; **(E)** IL-6; **(F)** IL-8; **(G)** IL-10; **(H)** TNF-α. NS: no sense; **P < 0.05.

After 2 weeks of colchicine treatment, a reduction in serum inflammatory factors was observed (Figure 3). Specifically, the concentration of IL-8 decreased from baseline by −28.65 (95% CI: −54.20 to −11.47, *p* = 0.0010), representing a percentage reduction of 65.5% (95% CI: −82.0% to −45.0%). Similarly, IL-6 levels decreased by −1.925 (95% CI: −4.510 to −0.29, *p* = 0.0028), reflecting a percentage change of −30.5% (95% CI: −54.0% to −5.0%; Table 2; Supplementary Figure 1).

**Table 2:** Changes in the levels of inflammatory factors before and after treatment with colchicine application.

Inflammatory factor	Before-treatment (median)	After-treatment (median)	Changing value	Percentage change	*p* value (Wilcoxon test)
IL-1B	2.045 (1.285, 2.605) (95%CI: 1.520 ~ 2.390)	1.615 (1.083, 3.000) (95%CI: 1.320 ~ 2.070)	−0.135 (−1.618, 1.288) (95%CI: −0.690 ~ 0.480)	−10.5% (−62.5% 89.25%) (95%CI: −38.00% ~ 30.00%)	0.7285
IL-2	1.895 (1.043, 2.550) (95%CI: 1.460 ~ 2.210)	1.320 (0.7825, 2.543) (95%CI: 1.050 ~ 1.630)	−0.380 (−1.405, 0.735) (95%CI: −0.860 ~ 0.220)	−20.5% (−62.75%, 46%) (95%CI: −45.00% ~ 18.00%)	0.0787
IL-4	1.370 (0.8675, 2.463) (95%CI: 1.080 ~ 1.780)	0.815 (0.3900, 1.395) (95%CI: 0.60 ~ 1.080)	−0.345 (−1.495, 0.5525) (95%CI: −1.040 ~ 0.01)	−34.5% (−78%, 69%) (95%CI: −58% ~ 1.0%)	0.0936
IL-5	1.270 (0.8850, 1.715) (95%CI: 1.110 ~ 1.510)	1.245 (0.6125, 1.635) (95%CI: 1.080 ~ 1.490)	−0.120 (−0.735, 0.530) (95%CI: −0.450 ~ 0.230)	−12% (−48.25%, 50.75%) (95%CI: −31.0% ~ 22.0%)	0.4534
IL-6	8.310 (3.965, 14.26) (95%CI: 5.340 ~ 10.35)	4.310 (2.563, 7.723) (95%CI: 3.520 ~ 6.080)	−1.925 (−10.25, 1.163) (95%CI:-4.510 ~ −0.29)	−30.5% (−73.75%, 28.25%) (95%CI: −54.0% ~ −5.0%)	0.0028
IL-8	61.51 (30.71, 189.6) (95%CI: 43.95 ~ 94.74)	15.92 (6.038, 53.74) (95%CI: 10.93 ~ 20.68)	−28.65 (−149.8, 7.163) (95%CI: −54.20 ~ −11.47)	−65.5% (−95%, 27%) (95%CI: −82.0% ~ −45.0%)	0.0010
IL-10	3.140 (2.383, 4.678) (95%CI: 2.640 ~ 4.060)	2.935 (1.988, 4.208) (95%CI: 2.270 ~ 3.350)	−0.57 (−1.565, 1.305) (95%CI: −1.070 ~ 0.770)	−14.5% (−48%, 53%) (95%CI: −37.0% ~ 32.0%)	0.3346
TNF-α	2.840 (2.045, 3.720) (95%CI: 2.310 ~ 3.320)	2.180 (1.400, 3.315) (95%CI: 1.730 ~ 2.860)	−0.29 (−1.575, 0.665) (95%CI: −1.030 ~ 0.09)	−14% (−47%, 33.5%) (95%CI: −36.0% ~ 4.0%)	0.1262

### 3.3 Secondary efficacy outcomes

**Figure 4:**
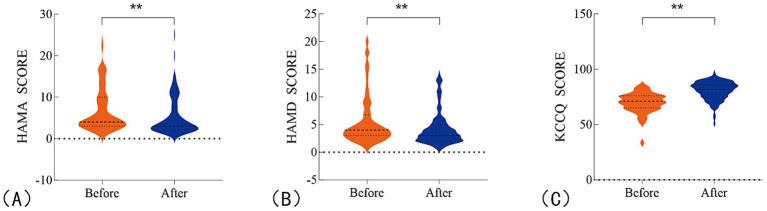
Changes in patients’ overall quality of life before and after treatment. **(A)** HAMA scores for anxiety; **(B)** HAMD scores for depression; **(C)** KCCQ scores for quality of life. **P <0.05.

For secondary endpoints, a significant improvement in patients’ overall quality of life was observed after 1 month of colchicine treatment, as compared to baseline (Figure 4). Specifically, the HAMA showed a change of −1 (95% CI: −1.0 to −1.0, *p* < 0.0001), with a percentage change of −25% (95% CI: −32.0% to −20.0%). Similarly, the HAMD exhibited a change of −1 (95% CI: −1.0 to 0.0, p < 0.0001), with a percentage change of −17% (95% CI: −25.0 to 0.0%). The KCCQ showed a change of 10 (95% CI: 9.0 to 12.0, p < 0.0001), with a percentage change of 14.5% (95% CI: 12.0 to 17.0%; Table 3; Supplementary Figure 2).

**Table 3:** Changes in quality of life before and after treatment with colchicine application.

Scale	Before –treatment (median)	After –treatment (median)	Descending value	Percentage of descend	*p* value (Wilcoxon test)
HAMA	4.000 (3.000, 10.000) (95%CI: 4.0 ~ 5.0)	3.000 (2.000, 7.750) (95%CI: 3.0 ~ 4.0)	−1 (−2, 0) (95%CI: −1.0 ~ −1.0)	−25% (−39.5%, 0%) (95%CI: −32.0% ~ 20.0%)	<0.0001
HAMD	4.000 (3.000, 6.750) (95%CI: 3.0 ~ 4.0)	3.000 (2.000, 5.000) (95%CI: 3.0 ~ 4.0)	−1 (−1, 0) (95%CI: −1.0 ~ 0.0)	−17% (−33%, 0%) (95%CI: −25.0% ~ 0.0%)	<0.0001
KCCQ	71.00 (65.00, 76.00) (95%CI: 69.0 ~ 72.0)	82.00 (75.00, 86.00) (95%CI: 80.0 ~ 84.0)	10 (6.25, 14.75) (95%CI: 9.0 ~ 12.0)	14.5% (9%, 21.75%) (95%CI: 12.0% ~ 17.0%)	<0.0001

HAMA, Hamilton Anxiety Scale; HAMD, Hamilton Depression Scale; KCCQ, Kansas City Cardiomyopathy Questionnaire.

### 3.4 Safety outcome

During the colchicine treatment period, the incidence of adverse reactions was observed to be 4.1%. Importantly, these adverse reactions were mild, with no serious adverse events reported. The most common side effects were gastrointestinal in nature, occurring in four patients, and primarily manifested as diarrhea and flatulence. Additionally, three patients experienced transient renal function abnormalities, characterized by a decrease in estimated glomerular filtration rate (eGFR) of more than 30 mL/min/1.73m^2^ from baseline. However, these patients did not show any clinical symptoms, and renal disease-related tests did not indicate any significant abnormalities. After appropriate dose adjustment, renal function returned to normal values in all affected patients.

No liver function impairment was observed in any of the participants throughout the study (Table 4). These findings suggest that colchicine is both safe and well-tolerated when used for the treatment of HFpEF, with adverse reactions being limited to mild gastrointestinal and transient renal changes, all of which were reversible with proper management.

**Table 4:** Safety endpoints of colchicine application.

Adverse reaction	Number of cases
Gastrointestinal manifestations	4
Diarrhea	2
Nausea	0
Flatulence	2
Gastrointestinal hemorrhage	0
Hepatotoxicity (AST or ALT > 3 times normal)	0
Nephrotoxicity (a ≥ 30% decline in eGFR)	0

AST, Aspartate aminotransferase; ALT, alanine aminotransferase; eGFR, estimated glomerular filtration rate.

## 4 Discussion

Our study demonstrated that inflammatory factors, particularly IL-8 and IL-6, were elevated in patients with early-stage HFpEF, consistent with findings from the Patrick study, which reported increased levels of IL-8, IL-6, and MCP-1 in the blood of 181 HFpEF patients (13–15). These findings further substantiate the role of inflammatory mediators in the pathogenesis of HFpEF and highlight the potential for targeting inflammation as part of the therapeutic approach (16–18).

Left ventricular diastolic dysfunction remains the hallmark pathophysiological feature of HFpEF. Previous research has shown that titin, a key regulator of myocardial passive tension, plays a central role in this dysfunction (19). Specifically, the transition of titin from the compliant N2BA isotype to the stiffer N2B isotype has been implicated in the development of diastolic dysfunction (20, 21). Our study suggests that IL-8, through binding to the CXCR1 and CXCR2 receptors on myocardial cells, may activate the phosphoinositide-specific phospholipase C pathway, leading to the activation of protein kinase C (PKC) (22, 23). This cascade ultimately results in the phosphorylation of titin, decreasing the N2BA: N2B ratio, reducing cardiomyocyte compliance, and contributing to left ventricular diastolic dysfunction (24). Colchicine, an anti-inflammatory agent, appears to mitigate this pathway by inhibiting IL-8 secretion, reducing titin phosphorylation, and restoring the balance of the N2BA: N2B ratio, thus improving myocardial stiffness and alleviating diastolic dysfunction (25).

Currently, only two randomized clinical trials have explored the effect of colchicine on heart failure. The study by Deftereos et al. showed that colchicine could reduce C-reactive protein (CRP) and IL-6 levels in patients with chronic stable heart failure but did not affect cardiac function or reduce hospital readmissions (26). Similarly, the COLICA trial by Pascual-Figal et al. found that colchicine reduced inflammatory markers such as CRP and IL-6 in patients with acute heart failure, but it did not significantly lower NT-proBNP levels, which is basically consistent with our research results (Supplementary Figure 3). Our study aligns with these findings, as we observed no significant difference in BNP levels before and after colchicine treatment, confirming that colchicine’s anti-inflammatory effects do not directly translate into immediate changes in cardiac biomarkers such as BNP (Supplementary Table 2) (27).

This study is the first to investigate the effects of colchicine on inflammatory responses in HFpEF patients. Colchicine significantly reduced IL-6 (~30%) and IL-8 (65%) levels, suggesting potential efficacy in modulating HFpEF-related inflammatory pathways. Assessment using HAMA, HAMD, and KCCQ indicated improvements in anxiety, depression, and cardiac-related quality of life (HAMA decreased by 25%, HAMD by 17%, and KCCQ increased by 15%), suggesting potential benefits beyond anti-inflammatory effects. Low-dose colchicine (0.5 mg daily) was well tolerated, with no serious adverse events or treatment discontinuations observed, indicating a favorable risk–benefit profile. These findings support colchicine as a potential anti-inflammatory intervention that may also improve quality of life in patients with HFpEF.

The safety data from this study indicate that colchicine was generally well tolerated, with a low incidence of adverse events. We specifically monitored gastrointestinal adverse events, including diarrhea, nausea, bloating, gastrointestinal bleeding, as well as liver and kidney function. The most common adverse effects observed were mild gastrointestinal symptoms, such as diarrhea and bloating. Based on the literature and clinical practice, the predefined discontinuation criteria included severe gastrointestinal discomfort, liver function abnormalities (ALT/AST ≥ 3 × upper limit of normal), and a decline in glomerular filtration rate (≥30%). In this study, no liver or kidney dysfunction was observed, and treatment did not lead to an increased discontinuation rate, even among elderly patients. These findings indicate that low-dose colchicine (0.5 mg daily) has a favorable safety profile and tolerability, making it a promising therapeutic option for patients with HFpEF.

### 4.1 Limitations

Despite these encouraging findings, several limitations of this study should be acknowledged. As a single-arm trial without a control group, definitive conclusions regarding the efficacy of colchicine in patients with HFpEF cannot be drawn. Future multi-center, randomized, double-blind, placebo-controlled trials are warranted to confirm the precise therapeutic effects of colchicine in this population. A subset of patients included in this study had mild to moderate coronary atherosclerosis (stenosis <50%) without evidence of acute ischemia or an indication for coronary intervention. Because coronary artery disease itself involves inflammatory processes, it may have influenced inflammatory biomarkers. Future studies should further exclude or stratify patients with coronary artery disease to better clarify its potential impact on the observed outcomes. Furthermore, although short-term improvements in symptoms and inflammatory markers were observed, assessments of quality of life and symptom burden relied primarily on total scores from HAMA, HAMD, and KCCQ, lacking detailed domain-specific analysis; thus, it remains unclear which aspects of psychological or functional status were most affected. Long-term follow-up is needed to evaluate the sustained impact of colchicine on HFpEF progression, patient-reported outcomes, and overall clinical prognosis. Future studies should also explore individualized treatment regimens based on patient characteristics to maximize therapeutic benefits while ensuring safety.

## 5 Conclusion

In conclusion, this study is the first to explore the use of colchicine for anti-inflammatory treatment in HFpEF patients. Our findings indicate that colchicine can safely and effectively reduce inflammatory factors such as IL-8 and IL-6. In addition, colchicine treatment was associated with improvements in patients’ quality of life and symptoms, as assessed by validated clinical scales. Further research is warranted to confirm these findings and to explore the underlying mechanisms, providing a potential therapeutic option for HFpEF.

## Data Availability

The raw data supporting the conclusions of this article will be made available by the authors, without undue reservation.
