# Supplementary figures and images for: Colchicine reduces inflammatory cytokines and improves symptoms in HFpEF: an observational pilot study
Source: Front Med (Lausanne). 2026 Jan 16;12:1702293. doi: 10.3389/fmed.2025.1702293 (PMC12857056; doi:10.3389/fmed.2025.1702293)

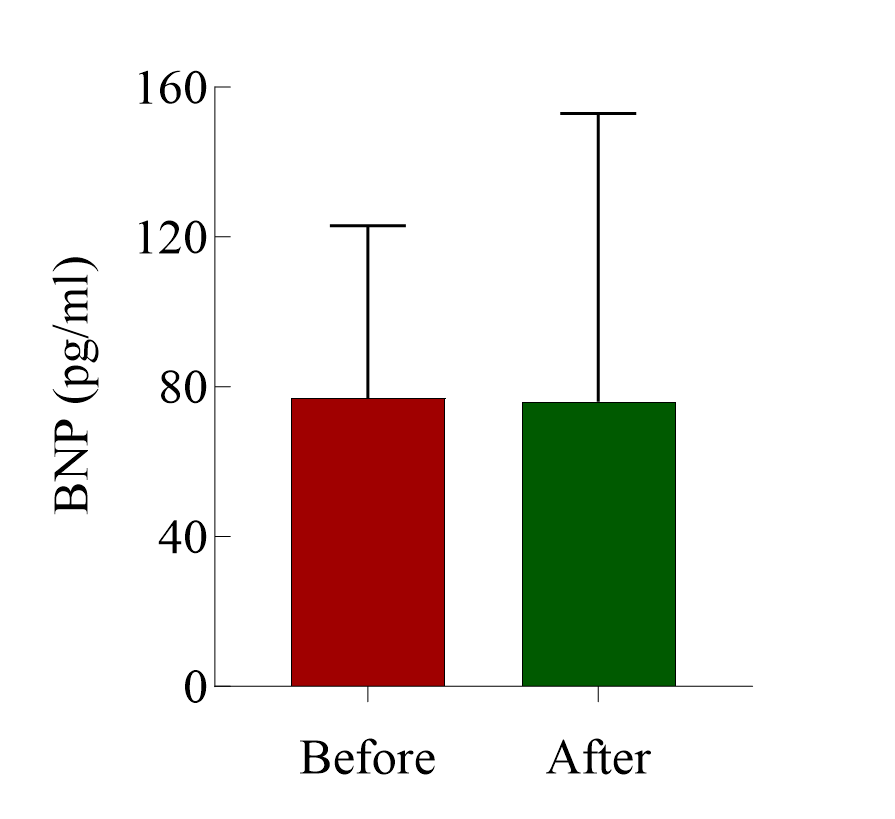

Supplement: Supplementary file 2 [file Image_2.TIF]
