# Supplementary material for: Colchicine reduces inflammatory cytokines and improves symptoms in HFpEF: an observational pilot study
Source: Front Med (Lausanne). 2026 Jan 16;12:1702293. doi: 10.3389/fmed.2025.1702293 (PMC12857056; doi:10.3389/fmed.2025.1702293)
Supplement: Supplementary file 4 [file Table_1.DOCX]

Table 1. Characterised by elevated levels of inflammatory factors at baseline

| Inflammatory factor | Case | Percentage（%） |
| --- | --- | --- |
| IL-1B | 3 | 2.78% |
| IL-2 | 2 | 1.85% |
| IL-4 | 14 | 12.96% |
| IL-5 | 4 | 3.70% |
| IL-6 | 60 | 55.56% |
| IL-8 | 89 | 82.41% |
| IL-10 | 27 | 25% |
| TNF-α | 6 | 5.56% |

Table 2. Changes in BNP before and after treatment with colchicine application

|  | Before -treatment  (median) | After -treatment  (median) | Descending value | Percentage of descend | *P* Value  (Wilcoxon Test) |
| --- | --- | --- | --- | --- | --- |
| BNP | 77.0（54.00,193.5）  （95%CI:58.0~123.0） | 76.0（44.5,212.5）  （95%CI:59.0~153.0） | -5.0（-29.0,76.5）  （95%CI:-19.0~-9.0） | -7%（-31.5%，54.0%）  （95%CI:-18.0%~22.0%） | 0.9139 |
